# Substance Abuse among Students in a Dental School

**DOI:** 10.31729/jnma.3900

**Published:** 2018-12-31

**Authors:** Shishir Bhatta, Manish Raj Sapkota, Sujita Shrestha, Rabindra Man Shrestha

**Affiliations:** 1 Department of Orthodontics, Kantipur Dental College, Basundhara, Kathmandu, Nepal; 2 Department of Community and Public Health Dentistry, Kantipur Dental College, Basundhara, Kathmandu, Nepal

**Keywords:** *alcohol*, *dental students*, *smoking*, *substance abuse*

## Abstract

**Introduction:**

Substance abuse has become a burning issue among the medical and dental students. Dental students, who later transform into dentists, have a significant role in substance abuse cessation. Thus the study was undertaken to quantify substance abuse among dental students of Kantipur Dental College.

**Methods:**

A descriptive cross-sectional study was conducted using pretested self-administered questionnaire among undergraduate and post graduate students of Kantipur Dental College. Convenience sampling was done and sample size was calculated.

**Results:**

Study revealed 166 (74.10%) as never smokers, 3 (1.30%) as former smokers and 55 (24.60%) as current smokers. Similarly 97 (43.3%) students never used alcoholic drink, 95 (42.41%) consumed alcohol monthly, 29 (12.95%) consumed alcohol 2–4 times a month and 3 (1.34%) consumed alcohol 2–3 times a week. A total of 78 (35%) students used cannabis.

**Conclusions:**

Substantial numbers of students were indulged in deleterious habits of smoking, tobacco and cannabis intake. Students need to be properly counselled to discourage substance abuse and create a healthy society.

## INTRODUCTION

Substance abuse is a burning issue in this modernized era. Medical students are indulged in abuse of stimulants (e.g. Cocaine), sedatives (e.g. Lorazepam), hallucinogens, e.g. Lysergic acid diethylamide (LSD), opioids, cannabis, alcohol and tobacco.^1,2^ Report of Global Health Professional Survey Nepal- 2006 shows, approximately 40 to 64% of students who participated in the survey had used tobacco during their life time, and about 17.4% to 23.7% were current smokers. A good proportion of students (58.0% dental and 43.5% medical) also reported nicotine dependency.^3^

Substance abuse is not only responsible for declined physical^4^ and mental fitness^5^ of an individual but it also increases the disability adjusted life in years (DALY) on long term.^6^

Dentists have a significant role in motivating their patients for substance abuse cessation. As the dental students are the one to transform into dentists, they represent a primary target for substance abuse cessation programs.

Thus the study was undertaken to quantify substance abuse among dental students of a Dental College.

## METHODS

This descriptive cross-sectional study was conducted among 250 dental (undergraduate and post-graduate) students of Kantipur Dental College from May 2017 to October 2017 after the ethical clearance from Institutional review committee (IRC) of KDC. Sample was collected using convenience sampling method. Sample size was calculated by the following formula:


$$\begin{array}{l} \text{Sample size}{\text{= Z}}^{2}\times \text{p}\times {\text{q/d}}^{2}\\ \text{Confidence Interval (CI) = 95\%}\\ \text{Error (d) = 6\%}\\ \text{Prevalence}(\text{P) =}21.57\\ \text{So, Sample size =}{\text{Z}}^{2}\times {\text{pq/d}}^{2}\\ =1.96\times 1.96\times 0.2157\times (1-0.2157)/(0{.06)}^{2}\\ =0.82863312\times 0.7843/0.0036\\ =180.52 \end{array}$$

Therefore, the calculated sample size was 181.

A total of 250 pretested, self-administered, structured questionnaire were distributed to the students which was collected after being filled. Written consent was taken prior to study and the participation was voluntary. Confidentiality of the participants was ensured. The participants were informed that their participation in the study was completely voluntary and they could withdraw from participation at any time. Study reveals tobacco, alcohol and cannabis as most commonly abused substances. So our study focuses in the abuse of these three substances. Smoking questionnaire contained details on tobacco smoking where the smokers were classified as: i. Never smoker ii. Former smoker iii. Current smoker^7^

Parental tobacco use was defined as habit of smoking tobacco by either or both parents.^8^ Also, use of cannabis was assessed. A modified Alcohol Use Disorder Identification Test (AUDIT) questionnaire was used to assess the magnitude and severity of alcohol abuse.^9^ It consisted 10 questions based on three domains which were categorized as hazardous alcohol use (pattern of alcohol consumption that increases the risk of harmful consequences for the users or others), harmful alcohol use (alcohol consumption that results in consequences to physical and mental health) and alcohol dependence (cluster of behavioral cognitive and physiological phenomena that develops after repeated alcohol use).^9^

A cut off score of eight was used, above which were suggestive of the indicators of hazardous and harmful alcohol use as well as possible alcohol dependence.

Questionnaires were administered during regular class in a voluntary manner, according to the protocol. The questionnaire was pretested among 25 students in order to ensure that the students could understand and answer the questions without any help. The validity of the questions was established by using standard questionnaire, which has been used in many previous studies across the world.^3,9^ Questionnaires were distributed to the participants by a single investigator and they were collected on the same day. Descriptive statistical analysis was performed using the SPSS version 20 software.

## RESULTS

Out of 250 questionnaires distributed, only 224 questionnaires could be recollected with an overall response rate of 89.6%, of which 186 (83%) of the respondents were female and 38 (17%) were male. Students who had experienced smoking (even one or two puffs) is shown in (Table 1).

**Table 1:** Response of students regarding cigarette smoking (even one or two puffs).

Gender	Smoking of cigarette (even one or two puffs)			Total
		Yes	No	
	female	64	122	186
	male	29	9	38
Total		93	131	224

**Figure 1.**
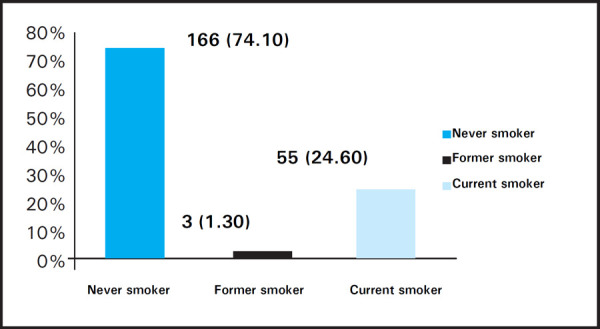
Smoking habit among the students n (%).

**Figure 2.**
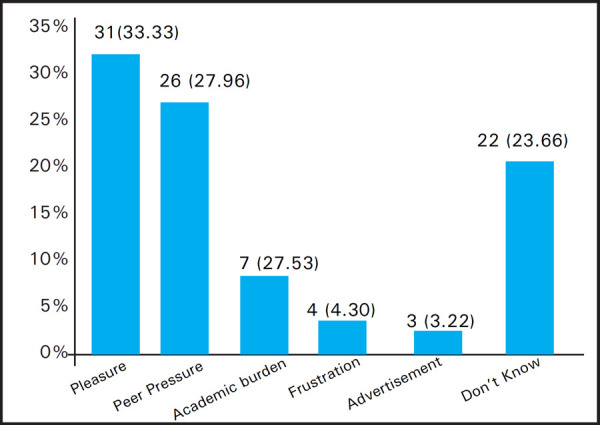
Causes of smoking among the students n (%).

Among those who smoked, 8 (8.60%) students first tried smoking at the age of 10–15 years, 47 (50.54%) at the age of 15–20 years and 38 (40.86%) when they were 20 years and above. The smoking habits were categorised as Never smoker, Former smoker and Current smoker which is depicted as below (Figure 1). Likewise, the cause of smoking is summarized in graph below (Figure 2).

With regard to cannabis intake, 78 (35%) respondents used cannabis out of which 53 (67.95%) were females and 25 (32.5%) were males.

**Figure 3.**
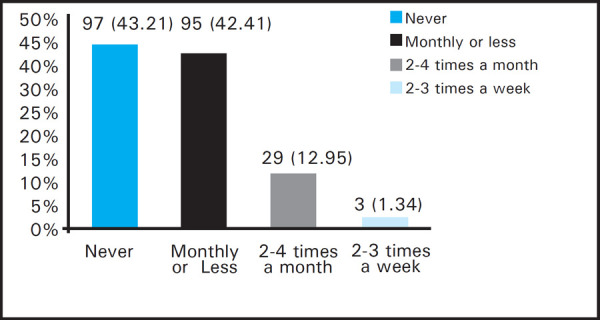
Frequency of Alcohol Consumption Among Dental Students.

On the other hand, 127 (56.7%) of the students had the habit of drinking alcohol. Synopsis of frequency of alcohol consumption is summarized below (Figure 3).

Harmful or hazardous alcohol use as well as incipience of alcohol dependence was found in 27 (12.05%) of total students.

## DISCUSSION

The study revealed that, among the total students enrolled 74.1% were non-smokers, 24.6% were current smokers and 1.3% were past smokers. The prevalence of cannabis use was 35% and alcohol based products was 55.8%. This is in agreement with the finding by Khanal et al where alcohol (57.6%) was the most prevalently used substance followed by tobacco (27.58%).^10^ Contrary to this, a study carried out among male medical and dental students by Ghimire et al showed that 38.4% students were smokers (46.4% occasional and 53.6% regular smokers) and 61.6% were non-smokers (87.8% never smokers and 12.2% ex-smokers). Nearly one third of the participants used to consume alcohol along with smoking.^3^ Additionally, a study on smoking and alcohol intake among medical students in south western region of Nepal revealed that 9% of the students were smokers and 20.33% were alcohol users.^11^ Similarly, a survey conducted to assess the alcohol use among physicians in a medical school in Nepal revealed 63% of the physician to be alcohol user.^12^ Differences seen in the prevalence of substance abuse may be attributed to the variation in definition and classification adopted for substance use, socio demographic, genetic as well as psychological parameters of the individual enrolled in the study.^13^

In current study, majority of the smokers started smoking when they were between 15 to 20 years of age (50.54%) which coincides with the finding of study conducted in BPKIHS and also with the study by Kumar et al which concluded that 62.7% of medical students started smoking between 18 to 20 years of age.^14^ Results suggesting the vulnerability of youth towards havoc of substance use advocate sincere efforts in protecting the youths as early as possible. The study depicted that the main cause of smoking among the students of KDC was pleasure (32.3%) followed by peer pressure (28%). A study by Budathoki et al to evaluate the substance use among third year medical students of Nepal concluded pleasure (40.9%) followed by irresistible desire (36.5%) and academic burden (20.4%) as the major cause of tobacco use.^15^ Similarly, Khanal et al suggested experimentation (42.3%) followed by stress relief (19.5%) and pleasure (15.4%) as culprits facilitating substance use.^10^ Parental smoking was reported by 10.7% of the respondents enrolled in the study which is significantly minimal compared to the findings by Ghimire et al (23.6%) and Kumar et al (27.7%).^3,14^

Alcohol use is globally being accepted as a social act. A study by Cozens concluded college students having higher prevalence of alcohol, drinking and alcohol disorder than non-college youth,^16^ which is equally applicable for medical student as well. In our study 12.1% of total students exhibited indicators of hazardous and harmful alcohol use as well as possible alcohol dependence, alcohol related harm (feeling of guilt after drinking, unable to recall last night event, alcohol inflicted injury, advised on alcohol cessation) already being experienced by 15.2% of total students. The results are in agreement with the findings by Budathoki et al where 16.6% of alcohol users required medical attention as a consequence of alcohol use like traffic accidents and intoxication.^15^

Since this is a self-administered questionnaire based study, there are chances of reporting and selection bias. Also the results may not be completely reliable as the responses were not validated by biomarkers of substance use. The study is carried out among limited number of students in a single institute; therefore the results cannot be generalized.

## CONCLUSIONS

The study reveals a substantial proportion of students indulged in substance abuse. Majority of the students used substance for sake of pleasure followed by irresistible peer pressure. Substance abuse by dental students has direct effect on their health as well as health of many people because they serve as a role model for their patients and communities. Therefore these students need to be properly guided and counselled in order to discourage such activities and create a substance free healthy society.

## Conflict of Interest

**None.**

## References

[1] Jaiswal HS, Jaiswal SS, Jain SL. (2017). Patterns of substance use in first year and final yearmedical students: a cross sectional study. Int J Recent Surg Med Sci...

[2] Ayala EE, Roseman D, Jeffrey SW, Mason HRC. (2017). Prevalence, perceptions and consequences of substance use in medical students. Med Educ Online..

[3] Ghimire A, Sharma B, Niraula SR, Devkota S, Pradhan PMS. (2013). Smoking habit among male medical and dental Students of B.P. Koirala Institute of Health Sciences.

[4] Sandvik L, Erikssen G, Thaulow E. (1995). Long term effects of smoking on physical fitness andlung function: a longitudinal study of 1393 middle aged Norwegian men for seven years. BMJ..

[5] Squeglia LM, Jacobus J, Tapert SF. (2009). The influence of substance use in adolescent braindevelopment. Clin EEG Neurosci..

[6] Whiteford HA, Ferrari AJ, Degenhardt L, Feigin V, Vos T. (2015). The global burdern of mental, neurological and substance use disorders: an analysis from the global burdern of disease study 2010.

[7] US Centres for Disease Control and Prevention (2010). Helath behavior of adults. United States, 2005-2007.

[8] Singh VV, Singh Z, Banerjee A, Basannar DR (2003). Determinants of smoking habits among medical students. MJAFI..

[9] Babor TF, Higgins-Biddle JC, Saunders JB, Monteiro MG. (2001). The alcohol use disorder identification test.

[10] Khanal P, Ghimire RH, Gautam B, Dhungana SK, Parajuli P, Jaiswal AK, Khanal B. (2010). Substance use among medical students in Kathmandu valley. J Nep Med Assoc..

[11] Jha SK, Agrawal R, Dhakal PK, Nepal M, Jayan A, Gautam N. (2014). Smoking and alcohol intake among students in medical college of south western region, Nepal. J Universal Col Med Sci..

[12] Kumar S, Pokharel B, Nagesh S, Yadav BK. (2006). Alcohol use among physicians in a medical school in Nepal. Kathmandu Univ Med J..

[13] Lutsky I, Hopewood M, Abram SE, Cerletty JM, Hoffmann RG, Kampine JP. (1994). Use of psychoactive substances in three medical specialties: anaesthesia, medicine and surgery. Can J Anaesth.

[14] Ganesh Kumar S, Subba SH, Unnikrishnan B, Jain A, Badiger S. (2011). Prevalence and factors associated with current smoking among medical students in coastal south India. Kathmandu Univ Med J..

[15] Budhathoki N, Shrestha MK, Acharya N, Manandhar A. (2010). Substance use among third year medical students of Nepal. J Nepal Health Res Counc..

[16] Firth Cozens J. (2001). Interventions to improve physicians' wellbeing and patient care. Soc Sci Med..

